# HSA-based multi-target combination therapy: regulating drugs’ release from HSA and overcoming single drug resistance in a breast cancer model

**DOI:** 10.1080/10717544.2018.1428245

**Published:** 2018-01-19

**Authors:** Yi Gou, Zhenlei Zhang, Dongyang Li, Lei Zhao, Meiling Cai, Zhewen Sun, Yongping Li, Yao Zhang, Hamid Khan, Hongbing Sun, Tao Wang, Hong Liang, Feng Yang

**Affiliations:** a State Key Laboratory for the Chemistry and Molecular Engineering of Medicinal Resources, Ministry of Science and Technology of China, Guangxi Normal University, Guilin, Guangxi, China;; b School of Pharmacy, Nantong University, Nantong, Jiangsu, China;; c Department of Biology, Southern University of Science and Technology, Shenzhen, Guangdong, China;; d Jiangsu Key Laboratory of Drug Discovery for Metabolic Disease, China Pharmaceutical University, Nanjing, Jiangsu, China

**Keywords:** Albumin, combination therapy, drug delivery systems, drug release, drug resistant

## Abstract

Multi-drug delivery systems, which may be promising solution to overcome obstacles, have limited the clinical success of multi-drug combination therapies to treat cancer. To this end, we used three different anticancer agents, Cu(BpT)Br, NAMI-A, and doxorubicin (DOX), to build human serum albumin (HSA)-based multi-drug delivery systems in a breast cancer model to investigate the therapeutic efficacy of overcoming single drug (DOX) resistance to cancer cells *in vivo*, and to regulate the drugs’ release from HSA. The HSA complex structure revealed that NAMI-A and Cu(BpT)Br bind to the IB and IIA sub-domain of HSA by N-donor residue replacing a leaving group and coordinating to their metal centers, respectively. The MALDI-TOF mass spectra demonstrated that one DOX molecule is conjugated with lysine of HSA by a pH-sensitive linker. Furthermore, the release behavior of three agents form HSA can be regulated at different pH levels. Importantly, *in vivo* results revealed that the HSA–NAMI-A–Cu(BpT)Br–DOX complex not only increases the targeting ability compared with a combination of the three agents (the NAMI-A/Cu(BpT)Br/DOX mixture), but it also overcomes DOX resistance to drug-resistant breast cancer cell lines.

## 1. Introduction

Increasing evidences have revealed that a single anticancer drug that inhibits a pathway is not sufficient to achieve tumor recession due to several reasons: (1) cancers are complicated diseases that involve multiple pathways (Hanahan & Weinberg, 2011); and (2) cancer cells often have intrinsic and acquired resistance to chemotherapeutic agents (Baguley, 2010; Gandin et al., 2013; Santini et al., 2014). Currently, multi-drug combination therapy has been adopted to overcome the deficiency of a single anticancer drug since several agents can simultaneously modulate different signaling pathways in diseased cells (Aw et al., 2013; Ma et al., 2013; Qi et al., 2016b; Huang et al., 2017).

Although the use of a combination of drugs has been promising for cancer therapy, major challenges accompany multi-drug combination therapy, including bioavailability, pharmacokinetics, and cellular uptake (Greco & Vicent, 2009; Parhi et al., 2012). These obstacles have limited the clinical success of combination therapy (Parhi et al., 2012). To overcome these challenges, several drug delivery systems have been explored to simultaneously deliver multiple drugs at the site of action and improve anti-tumor activities (Kurapati & Raichur, 2012; Liu et al., 2012; Parhi et al., 2012; Yan et al., 2012; Bao et al., 2016; Qi et al., 2016b; Li et al., 2017; Yang et al., 2018). Among them, human serum albumin (HSA)-based multi-drug systems are promising owing to HSA’s unique properties relative to other drug carriers (Furukawa et al., 2011; Kratz & Elsadek, 2012; Kratz, 2014; Yang & Liang, 2015). Interestingly, to avoid possible mutual interference of several anticancer drugs within a single carrier, Yang group designed HSA-based multidrug delivery systems by rational regulating their spatial distribution in HSA (Qi et al., 2016b).

The previous studies have revealed that the HSA carrier is helpful to overcome cancer cells’ resistance to a single agent (Garmann et al., 2008). Thus, we not only designed HSA-based multi-drug systems to improve the efficiency of multi-drug combination therapy *in vivo*, but also to enhance the capacity of overcoming cancer cells’ resistance to a single agent. We needed to consider two potential problems: (1) if the drug that is conjugated or bound to HSA is weak, the drug will be released from the HSA carrier into the bloodstream, leading to unexpected side effects *in vivo*; and (2) if the drug that is conjugated or bound to HSA is tight, the drug will not be released from the HSA carrier into the cancer cells. Thus, while we need rational designed HSA-based multi-drug systems to increase drugs’ delivery efficiency, we should regulate their release from HSA *in vivo*.

**Figure 1.**
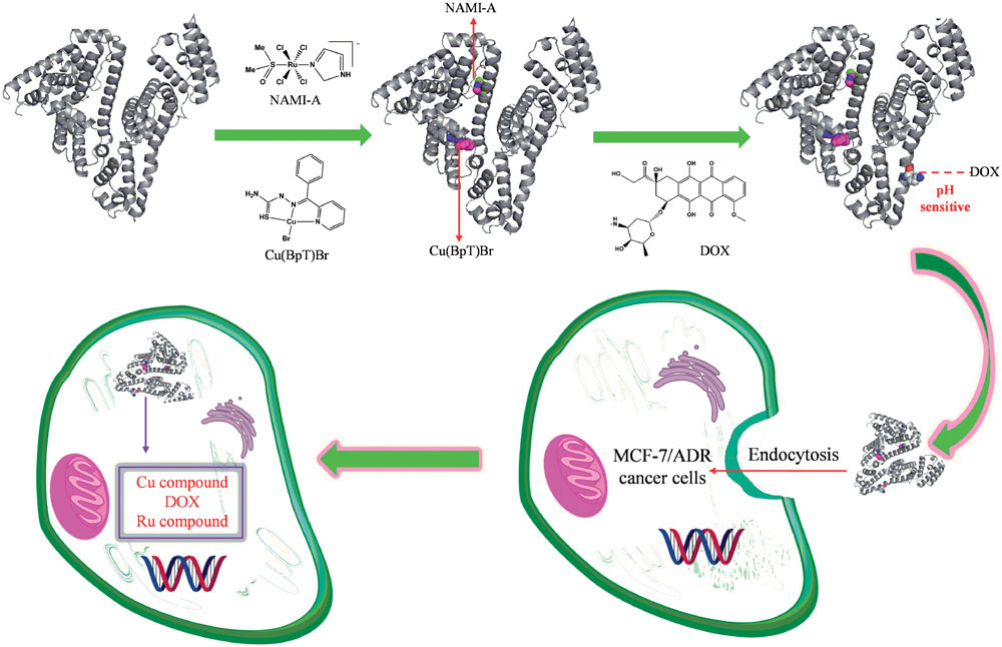
Hypothesis of establishing HSA co-deliver three anticancer agent based on the nature of HSA and agents.

**Figure 2.**
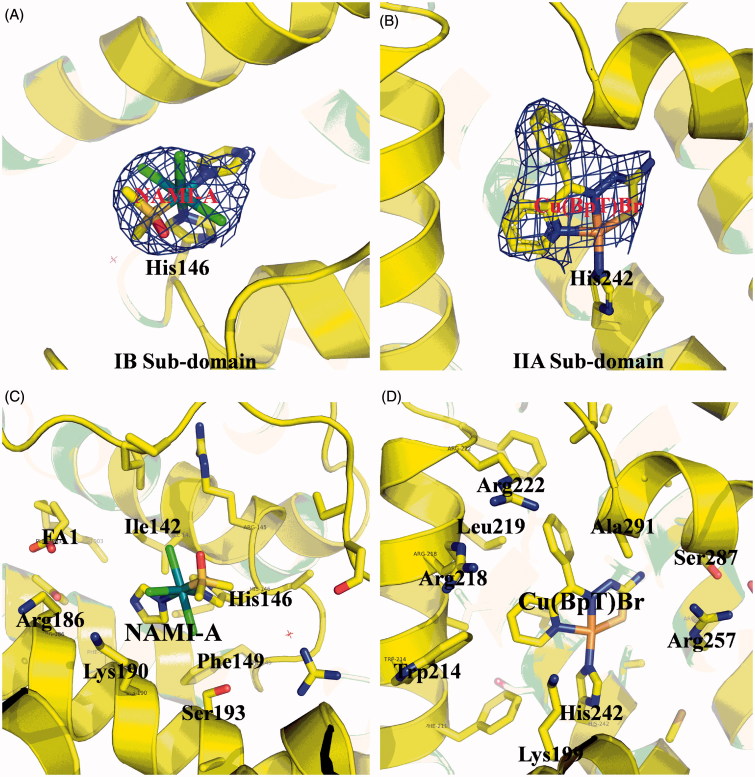
(A) and (B) Experimental sigmaA weighted 2Fo-Fc electron density map of Ru and Cu compounds at IB subdomain and IIA subdomain of HSA, respectively. (C) and (D) Structural binding environment of Ru and Cu compounds at IB subdomain and IIA subdomain of HSA, respectively. The amino acid chains that are close to the drug molecules are shown as sticks.

Breast cancer, which is very common, is responsible for a large number of cancer deaths among women worldwide (Torre et al., 2015). Although doxorubicin (DOX) is the first-line drug used to treat breast cancer, cancer cells have acquired resistance to it (Wong et al., 2006; Cao et al., 2015). Ruthenium and copper agents have been promising next-generation metal agents for the treatment of various cancer with unique anticancer mechanisms (Ruiz-Azuara & Bravo-Gomez, 2010; Santini et al., 2014; Bergamo & Sava, 2015). Interestingly, NAMI-A (imidazolium *trans*-imidazoledimethylsulphoxide-tetrachlorido ruthenate) in combination with many drugs shows more effective than individual treatments (Bergamo & Sava, 2015; Bergamo et al., 2015). In addition, Cu agents containing thiosemicarbazide ligands offer a different spectrum of anticancer activity and the prospect of non-cross-resistance (Beraldo & Gambino, 2004; Santini et al., 2014; Park et al., 2016). Taking into consideration the above factors, based on HSA’s binding properties for the drugs, we used three different anticancer agents, 2-benzoylpyridine thiosemicarbazone copper(II) [Cu(BpT)Br], NAMI-A, and DOX, which work on different target sites (Sava et al., 1998; Mizutani et al., 2003; Sava et al., 2003; Santini et al., 2014), to build a mice model of HSA-based multi-drug combination therapy by conducting the following studies: (1) we constructed HSA delivery systems for a combination of three agents (Figure 1); (2) we regulated the release behavior of the three agents from HSA; and (3) we confirmed the feasibility of the combination of the three agents and HSA multi-drug delivery systems to overcome cancer cells’ resistance to DOX *in vivo*.

## 2. Materials and methods

HSA and DOX were purchased from the Sigma Chemical Company (St. Louis, MO). Cu(BpT)Br and NAMI-A were synthesized according to reported methods (Adigun et al., 2014; Qi et al., 2016b). All of the other solvents and chemicals used were of high purity and available from commercial sources. Culture medium DMEM, fetal bovine serum (FBS), antibiotice–antimycotic and phosphate-buffered saline (PBS) came from E.U. Gibco BRL (Carlsbad, CA). Normal lung fibroblast cells WI-38, human breast cancer cell line MCF-7, and drug-resistant MCF-7/ADR cells were purchased from the American Type Culture Collection and the German Collection of Microorganisms and Cell Cultures. The cells were maintained in DMEM supplemented with 50 mg/mL of streptomycin, 10% FBS, and 50 U/mL of penicillin at 37 °C and 5% CO_2_.

### 2.1. X-ray crystallography of HSA–NAMI-A–Cu(BpT)Br complex

HSA was purified according to a previous method (Curry et al., 1998). To prepare the HSA complex, we mixed 100 µL HSA (100 mg/mL), 380 µL 2.5 mM palmitic acid (PA), 45 µL Cu(BpT)Br (10 mM), and 45 µL of NAMI-A (10 mM) overnight. Then, the mixtures were concentrated to 100 mg/mL with a Millipore spin filter (10,000 Da cutoff). HSA complex crystals were grown using sitting drop vapor diffusion according to the following procedures (Gou et al., 2015a; Qi et al., 2016c; Gou et al., 2017). We mixed 1 µL HSA complex with an equal volume of reservoir solution containing 28–32% (w/v) polyethylene glycol 3350, 50 mM potassium phosphate (pH 7.5), 5% glycerol, and 4% DMSO. HSA complex crystals were frozen in liquid nitrogen when the crystals were picked from solution.

We used the beamline BL17U in the Shanghai Synchrotron Radiation Facility to collect X-ray diffraction data at 100 K (Wang et al., 2015). The HSA complex data were integrated and scaled by HKL2000 (Otwinowski & Minor, 1997) (Table S1). HSA complex structures were resolved by molecular replacement in CCP4i (Krojer et al., 2017), and they were refined in the PHENIX program according to the reported procedure (Adams et al., 2010) (Table S1). The HSA complex structure figures were depicted by PyMOL software (DeLano, 2004).

### 2.2. Synthesis and characterization of HSA–DOX or HSA–NAMI-A–Cu(BpT)Br–DOX complex

The conjugation of DOX to HSA or the HSA–NAMI-A–Cu(BpT)Br complex using a *cis*-aconityl bond was prepared as previously reported (Shen & Ryser, 1981; Yoo et al., 2002; Du et al., 2013). In brief, doxorubicin hydrochloride (7 mg) was dissolved in water (4 mL), and then a 1,4-dioxane (200 µL) solution of *cis*-aconitic anhydride (5 mg) was slowly added to the doxorubicin hydrochloride solution with stirring. The reaction mixture was immediately adjusted to pH 9.0, and then the mixture carried out in an ice bath. After 30 min, the pH was adjusted to 7.0 and the mixture was stirred for another 30 min. We slowly added 1 M HCl to the mixture until the *cis*-aconitic anhydride-doxorubicin heavy precipitate was formed. After 30 min on ice, the precipitate was recovered by centrifugation (8000 rpm, 15 min). Next, the *cis*-aconitic anhydride-doxorubicin (4.2 mg, 6 µM), N-hydroxy-succinimide (NHS, 2 mg), and 1-ethyl-3-(3-dimethylaminopropyl) carbodiimide (EDCI, 3.5 mg) were dissolved in distilled water (3 mL) and stirred at room temperature in the dark for 12 h. Finally, the solution was mixed with HSA or the HSA–NAMI-A–Cu(BpT)Br complex (20 mL of 17 mg/mL in distilled water, 5 µM), and stirred for another 24 h at room temperature in the dark. After the reaction, the solution was purified and separated from the free *cis*-aconitic anhydride-doxorubicin using Sephadex G-25. The coupling ratio of *cis*-aconitic anhydride-doxorubicin to HSA or the HSA–NAMI-A–Cu(BpT)Br complex was determined by UV–vis spectrometry.

Matrix-assisted laser desorption ionization time-of-flight mass spectrometry (MALDI-TOF-MS) was used to determine whether DOX was conjugated to HSA or the HSA–NAMI-A–Cu(BpT)Br complex. The samples of HSA, HSA–DOX, HSA–NAMI-A–Cu(BpT)Br, and HSA–NAMI-A–Cu(BpT)Br-DOX were prepared using the dried droplet method with fresh 10 mg/mL sinapinic acid as the matrix solution. The protein sample solution (100 μL, a series of 1:10 dilutions) was mixed on the target with the matrix solution (100 μL) and allowed to air-dry. The MALDI-TOF-MS data were recorded in the *m/z* 30,000 − 100,000 range in a positive linear mode.

### 2.3. Release behavior of three agents from the HSA complex

To evaluate the release behavior of the three agents from the HSA complex, *in vitro* release profiles of the three agents from the HSA–NAMI-A–Cu(BpT)Br-DOX complex were tested at different pH levels (4.7 and 7.4). In brief, 5 mL HSA complex were dialyzed in a tube containing 50 mL of pH 4.7 and pH 7.4 buffers for 48 h. The amount of NAMI-A and Cu(BpT)Br released from the HSA complex was determined with a graphite furnace atomic absorption spectrometer (GF-AAS). The amount of DOX released from the HSA complex was calculated by UV–vis spectrometry.

### 2.4. *In vitro* anticancer activity

The 3-(4,5-dimethylthiazol-2-yl)-2,5-diphenyltetrazolium bromide (MTT) experiment has been performed according to a published method (shown in Supporting Information) (Qi et al., 2016a,b).

### 2.5. *In vivo* animal studies

The MCF-7/ADR tumor-bearing mice (40) were randomly divided into five groups when the tumor volume was approximately 80 mm^3^ so they could be used in the antitumor activity study. The mice in different treatment groups were intravenously injected with NaCl, DOX (at a dose of 6 µmol per kg body weight), the three-agent combination [DOX (2 µmol/kg) + NAMI-A (2 µmol/kg) + Cu(BpT)Br (2 µmol/kg)], HSA–DOX (6 µmol per kg body weight), and HSA–NAMI-A–Cu(BpT)Br–DOX (2 µmol per kg body weight) every 3 d. All of the mice in all of the groups were earmarked and followed individually throughout the experiments. The length and the width of the tumor and the body weights of the mice were measured before every injection and at the end of the experiment. The volume was calculated using the following equation: tumor volume (V) = 1/2 × width^2^ × length. Mice were killed after 21 d of treatment, and the major organs and tumor tissues of mice were placed in a Teflon container and mineralized in a microwave oven under pressure (system Milestone MSL 1200) in 30% hydrogen peroxide (1 mL) and in the presence of 7 mL of concentrated HNO_3_. Finally, inductively coupled plasma-atomic emission spectrometry (ICP-AES) was used to measure the Cu content in the major organs and tumors. In addition, major organs (heart, kidney, and liver) and tumor tissues were excised for histopathological analysis with terminal deoxynucleotidyl transferase dUTP nick end labeling (TUNEL) assay hematoxylin and eosin (H&E) staining.

### 2.6. Statistical analysis

Statistical analysis was performed using Student’s *t* test to compare experiment results. Results were expressed as the mean ± SD and considered to be significant when *p* < .05.

## 3. Results

### 3.1. Feasibility of establishing HSA-based multi-drug delivery systems

We used X-ray crystallography and MALDI-TOF-MS spectra to determine whether different drugs could bind to different areas of the HSA carrier. The electron density map of the compounds in the HSA complex clearly show one NAMI-A molecule and one Cu(BpT)Br molecule bound to the IB and IIA subdomains, respectively (Figure 2(A,B)). The overall structure of the HSA–NAMI-A–Cu(BpT)Br complex is heart shaped. In the HSA IB subdomain, NAMI-A binds in a long and narrow cavity, and has primary hydrophobic interactions with the surrounding residues, including Ile142, His146, Phe149, Leu154, Phe157, Tyr161, Arg186, Gly189, Lys190, and Ser193 (Figure 2(C)). His146 replaces the Cl ligand and coordinates to the Ru(III) center of NAMI-A (Figure 2(A)). In the HSA IIA subdomain, Cu(BpT)Br binds to a large hydrophobic pocket delimited by residues, including Ala291, Ser287, His242, Trp214, Leu260, Arg218, Arg222, Lys199, Leu219, Phe223, Leu238, Arg257, Ile264, Ile290, and Leu234 (Figure 2(D)). His242 coordinates to the Cu center of Cu(BpT)Br by replacing the Br ligand of Cu(BpT)Br (Figure 2(B)).

**Figure 3.**
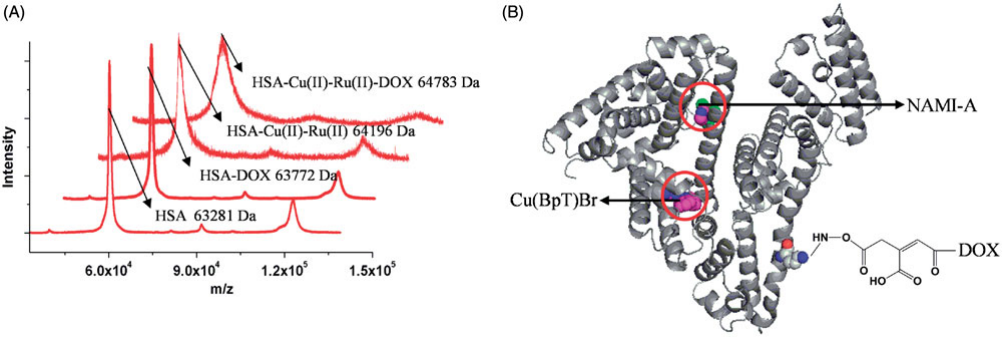
(A) The MALDI-TOF-MS spectrum of HSA and HSA complexes. (B) The model of HSA–NAMI-A–Cu(BpT)Br–DOX complex.

The MALDI-TOF-MS spectrum showed an increase in molecular weight of approximately 600 Da for the HSA–Cu(II)–NAMI-A–DOX complex relative to HSA–NAMI-A–Cu(BpT)Br, corresponding to the molecular weight of *ca.* one DOX molecule was tethered per each HSA–NAMI-A–Cu(BpT)Br molecule (Figure 3(A)), implying that the HSA–NAMI-A–Cu(BpT)Br–DOX complex is established (Figure 3(B)).

### 3.2. Release behavior of the three agents from the HSA complex

**Figure 4.**
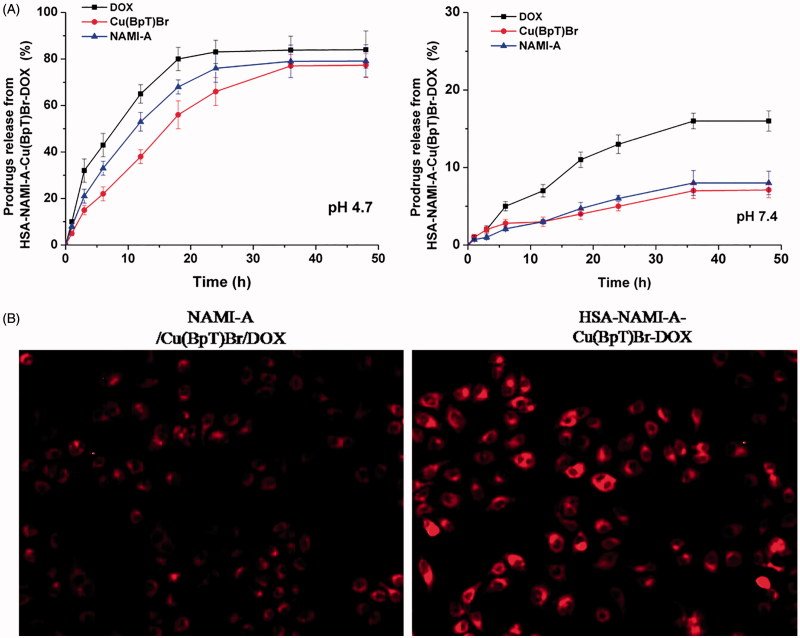
(A) The profiles of DOX or NAMI-A or Cu(BpT)Br release from HSA–NAMI-A–Cu(BpT)Br-DOX complex at different pH (citric-phosphate buffer). Results are the mean ± SD (*n* = 3): **p < .01. (B) Fluorescence microscope images of MCF-7/ADR cells. MCF-7/ADR cells treated with 10 μM three-agent combination (NAMI-A/Cu(BpT)Br/DOX) and 10 μM HSA–NAMI-A–Cu(BpT)Br–DOX complex for 5 h, respectively.

The amount of DOX released from the HSA–NAMI-A–Cu(BpT)Br–DOX complex at pH 4.7 was approximately 90%, and the amount of DOX released from HSA complex was 16% at pH 7.4 (Figure 4(A)). Approximately 5% of the NAMI-A or Cu(BpT)Br was released from the HSA–NAMI-A–Cu(BpT)Br–DOX complex within 48 h in the pH 7.4 buffer, whereas up to 80% of NAMI-A or Cu(BpT)Br was released from the HSA-Cu(II)-Ru(III)-DOX complex in the pH 4.7 buffer (Figure 4(A)).

### 3.3. Capacity of the HSA–NAMI-A–Cu(BpT)Br–DOX complex to overcome cancer cells’ resistance to DOX *in vitro*

To evaluate whether the HSA–NAMI-A–Cu(BpT)Br–DOX complex overcome the resistance of MCF-7 breast cancer cells to DOX, we investigated the cytotoxicity of the HSA–NAMI-A–Cu(BpT)Br–DOX complex to MCF-7/ADR and MCF-7 breast cancer cells. The IC_50_ dose of DOX for the resistant MCF-7/ADR cells was significantly greater than that of DOX for the sensitive MCF-7 cells (Table 1). However, the HSA–NAMI-A–Cu(BpT)Br–DOX complex and the three-agent combination have high cytotoxicity to MCF-7/ADR and MCF-7 cells, especially for HSA–NAMI-A–Cu(BpT)Br–DOX complex. Obviously, *in vitro* data demonstrated that HSA–NAMI-A–Cu(BpT)Br–DOX complex can effectively overcome the resistance of MCF-7/ADR cells compared with DOX.

**Table 1.** Inhibition of human cancer cell lines growth (IC_50_, μM) for agent, agents combination and HSA–NAMI-A–Cu(BpT)Br–DOX complex.

	Antitumor activity IC_50_ (μM)		
Compound	MCF-7	MCF-7/ADR	WI-38
Cu(BpT)Br	3.91 ± 0.21	4.25 ± 0.41	4.80 ± 0.38
NAMI-A	>50	>50	>50
DOX	5.85 ± 0.38	>50	5.17 ± 0.53
DOX/NAMI-A	5.77 ± 0.41	>50	5.11 ± 0.47
DOX/Cu(BpT)Br	2.41 ± 0.22	3.82 ± 0.31	1.89 ± 0.24
Cu(BpT)Br/NAMI-A	3.86 ± 0.33	4.17 ± 0.37	4.69 ± 0.48
NAMI-A/Cu(BpT)Br/DOX	2.32 ± 0.21	3.41 ± 0.24	1.72 ± 0.15
HSA–NAMI-A–Cu(BpT)Br–DOX	1.43 ± 0.09	1.58 ± 0.08	1.67 ± 0.19

Interestingly, by incubating of MCF-7/ADR cells with HSA–NAMI-A–Cu(BpT)Br–DOX complex, the DOX florescence signals were stronger than that of MCF-7/ADR cells incubated with three-agent combination, implying that HSA facilitate to enhance uptake of MCF-7/ADR cells for three-agent combination (Figure 4(B)).

### 3.4. Animal studies of the HSA–NAMI-A–Cu(BpT)Br–DOX complex

To further evaluate the therapeutic efficacy of the HSA–NAMI-A–Cu(BpT)Br–DOX complex for DOX resistance (MDR) tumors *in vivo*, the breast cancer MCF-7/ADR xenograft mouse model was established.

#### 3.4.1. Capacity of the HSA–NAMI-A–Cu(BpT)Br–DOX complex to overcome cancer cells’ resistance to DOX *in vivo*

**Figure 5.**
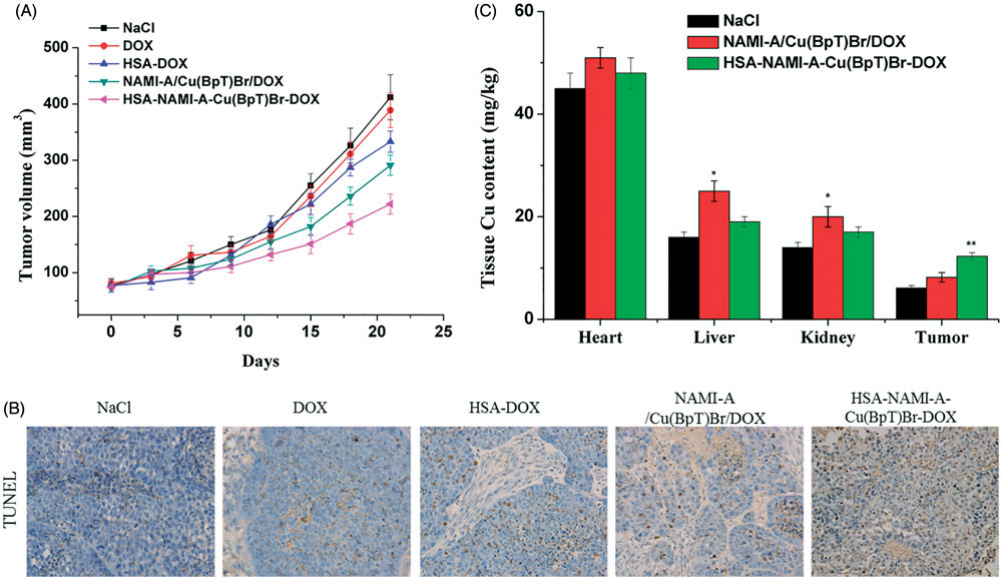
*In vivo* anti-tumor activity of HSA–NAMI-A–Cu(BpT)Br–DOX, three-drug combination, HSA-DOX, free DOX, and NaCl (*n* = 6). (A) Tumor volumes of MCF-7/ADR-bearing mice as a function of time. (B) Apoptotic cells were evaluated in tumor tissue using a TUNEL assay. (C) Tissue copper of MCF-7/ADR tumor-bearing nude mice after treatment with saline, three-drug combination and HSA–NAMI-A–Cu(BpT)Br–DOX. Results are the mean ± SD (*n* = 3): *p < .05; **p < .01.

Compared with the control group, the tumor volume after 21 d of treatment was 88.9 ± 9.1% for DOX, 62.8 ± 5.8% for the three-agent combination, 68.9 ± 7.2% for HSA-DOX, and 49.6 ± 5.3% for the HSA–NAMI-A–Cu(BpT)Br–DOX complex (Figure 5(A)). Compared with the control group, the tumor inhibitory rate (TIR) of DOX was *ca.* 7.2%, HSA-DOX was *ca*. 21.5%, three-agent combination was *ca*. 24.6%, and the HSA–NAMI-A–Cu(BpT)Br–DOX complex was *ca.* 44.8%.

Furthermore, the TUNEL-stained tissue sections treated with NaCl, DOX, HSA–DOX, three-agent combination, and HSA–NAMI-A–Cu(BpT)Br–DOX showed obvious differences in tumor tissue morphology. The results in Figure 5(B) showed that the tumor treated with DOX exhibits a similar morphology to that of the control group. However, tumors treated by HSA–DOX, three-agent combination and HSA–NAMI-A–Cu(BpT)Br–DOX complex showed increasing apoptosis compared with tumor treated by control or DOX group (Figure 5(B)). In particular, HSA–NAMI-A–Cu(BpT)Br–DOX complex was more effective in promoting tumor cell necrosis than HSA-DOX and three-agent combination.

#### 3.4.2. Targeting ability of the HSA–NAMI-A–Cu(BpT)Br–DOX complex *in vivo*

To compare the targeting ability of HSA-based multi-drugs delivery systems with three-agent combination, we used ICP-AES to determine the Cu content in the tumor tissues and main organs because HSA-based multi-drugs delivery systems can deliver three agents into cancer cells at the same time. Thus, we measured the copper content in the tumors of mice treated with the three-agent combination and the HSA–NAMI-A–Cu(BpT)Br–DOX complex. The ICP-AES data showed that there was more Cu in the MCF-7/ADR tumors treated by the HSA–NAMI-A–Cu(BpT)Br–DOX complex than in the tumors treated with the three-agent combination (Figure 5(C)). Furthermore, our data revealed that HSA helps to decrease the accumulation of drugs in other major organs (Figure 5(C)).

In addition, agent-related side effects and toxicities to major organs were examined by H&E staining (Figure S1). There were no abnormalities observed in any of the heart sections. Damage to the liver (inflammatory cell infiltration and hepatocyte edema), and kidneys (renal epithelial cells vacuolar degeneration) was observed in mice treated with the DOX and the three-agent combination. This damage was decreased in mice treated with HSA–NAMI-A–Cu(BpT)Br–DOX complex.

## 4. Discussion

For the multi-drug combination therapy for cancer, we should not only enhance the multi-drug targeting ability, but also render the drugs able to enter cancer cells at the same time. HSA-based multi-drug delivery systems may be one of the most promising strategies to achieve the above objectives because of HSA’s unique properties (Qi et al., 2016b). Thus, how to rationally construct HSA-based multi-drug delivery systems based on the nature of HSA is a challenge. While we can design pro-drugs with groups reacting to special residues of HSA, such as cysteine and lysine, we can form the HSA complex with drugs that directly bind to HSA (Stehle et al., 1997; Kratz, 2007; Kratz, 2008; Hanif et al., 2012; Gou et al., 2015a; Gou et al., 2016a,b; Qi et al., 2016a). HSA has three main binding sites for various kinds of endogenous and exogenous compounds: site 1 in the IIA sub-domain, site 2 in the IIIA sub-domain, and site 3 in the IB sub-domain (Zsila, 2013). Among the three binding sites, the endogenous non-esterified fatty acids (FA) occupy site 2 or displace drugs to bind to site 2 *in vivo* because site 2 is the strongest binding site of FA (Simard et al., 2005, 2006; Yang et al., 2013). Thus, reasonable consideration of the nature of drugs has resulted in our development of HSA multi-drug delivery systems in which two agents, respectively, bind to the IB and IIA sub-domains of HSA while the third drug is conjugated to HSA (Figure 1). Based on the structure of HSA–Cu(BpT)Br complex, Cu(BpT)Br binds to the IIA sub-domain of HSA by His146 specific coordinated with Cu center. However, Ru agents may bind to the IB and/or IIA sub-domain(s) of HSA because the binding site and binding mode of metal agents to IIA sub-domain depend on their molecular structure (Webb et al., 2013; Zhang et al., 2014; Ferraro et al., 2015). Therefore, the optimal strategy for HSA delivering two metal agents is that Cu(BpT)Br and NAMI-A bind to the IIA and IB sub-domains of HSA, respectively. Indeed, our results fit well with our hypothesis. NAMI-A and Cu(BpT)Br bind to the IB and IIA sub-domains of HSA, respectively, and one DOX molecule is conjugated to the HSA-NAMI-A-Cu(BpT)Br (Figures 2 and 3). Obviously, the HSA delivery system that we constructed can co-deliver three drugs to reach cancer cells at the same time.

To prevent drugs from being released from HSA into the blood stream and instead have the drugs released from HSA inside the cancer cells, determining how to regulate the drugs’ releasing behavior from the HSA carrier *in vivo* is important and necessary. Thus, we designed two metal compounds that bind to the IB and IIA sub-domains of HSA, because N-donor residue can replace a leaving group of metal compounds that coordinate to the metal ions (Figure 2), and then we tethered the third agent to HSA by designing a chemical linker that is sensitive to the acidic environment so that it reacts to lysine residues (Figure 3). Our results showed that a small amount of DOX was released from HSA, and a limited amount of metal compounds was released from the HSA carrier at pH 7.4 (Figure 4). In contrast, up to 90% of DOX was released from HSA, and *ca.* 80% of metal compounds were released from the HSA complex in pH 4.7 buffer (Figure 4). The releasing profile suggested that the HSA multi-drug delivery systems would be stable in the blood during *in vivo* circulation and that the three agents would be released after accumulating in the acidic lysosomes of cancer cells.

Our results revealed that HSA–NAMI-A–Cu(BpT)Br–DOX can overcome cancer cell resistance to DOX to some extent, which again confirmed previous studies in which a multi-drug combination overcome cancer cell resistance to a single drug (Chen et al., 2017; Kayani et al., 2018; Wang et al., 2017). Importantly, the tumor inhibitory rate of HSA–NAMI-A–Cu(BpT)Br–DOX (*ca.* 44.8%) is about two-fold that of the three-agent combination (*ca.* 24.6%) and HSA-DOX (*ca.* 21.5%). Obviously, HSA improved the efficacy of three-agent combination for overcoming cancer cells’ resistance to DOX *in vivo*. Indeed, the mechanism of resistance of cancer cells against drug is complicated (Seebacher et al., 2016). Such as, the efflux pump is an important mechanism (Huang et al., 2014; Seebacher et al., 2016). P-glycoprotein can use the energy from ATP-hydrolysis to pump free small-molecule anticancer drug out of tumor cells, resulting in a reduction of the drug accumulation in tumor cells (Gottesman et al., 2002). Therefore, we speculated that the HSA, as a nanocarrier, may bypass the P-glycoprotein efflux pump and accumulate HSA complex into MCF-7/ADR cancer cells (Chavanpatil et al., 2007; Iversen et al., 2011; Huang et al., 2014).

Importantly, our results showed that HSA–NAMI-A–Cu(BpT)Br–DOX complex decrease side effects relative to three-agent combination, because HSA facilitates more agents targeting accumulating into tumors *via* the enhanced permeability and retention (EPR) effect confirmed by other groups (Maeda et al., 2001; Iyer et al., 2006; Torchilin, 2011) (Figure S2 and 5(C)). In addition, the tumor endothelium expresses two albumin-binding proteins SPARC and gp60 receptor, which may facilitate the uptake and retention of the HSA complex in the tumor interstitium (Kratz, 2014; Gou et al., 2015b). Together, our results provide a novel approach for optimizing the capacity of overcoming cancer cell resistance to a single anticancer agent through the rational design of multi-drug delivery systems.

## 5. Conclusions

Based on the nature of anticancer agents and HSA, we proposed and constructed HSA-based multi-drug delivery systems, regulated the drugs’ releasing behavior from HSA, and overcame MCF-7/ADR cancer cells’ resistance to DOX *in vivo*. The HSA–NAMI-A–Cu(BpT)Br–DOX complex can selectively accumulate at the tumor site relative to the unregulated delivery of a three-drug combination. An HSA-based multi-drug delivery system may represent an innovative method to overcome cancer cells’ resistance to a single agent and target combination therapy for cancer.

## Supplementary Material

IDRD_Yang_et_al_Supplemental_Content.docx
