# Supplementary material for: HSA-based multi-target combination therapy: regulating drugs’ release from HSA and overcoming single drug resistance in a breast cancer model
Source: Drug Deliv. 2018 Jan 19;25(1):321–9. doi: 10.1080/10717544.2018.1428245 (PMC6058715; doi:10.1080/10717544.2018.1428245)
Supplement: IDRD_Yang_et_al_Supplemental_Content.docx [file IDRD_A_1428245_SM5916.docx]

**Supporting Information**

**1. Materials and Methods**

***1.1 Crystal structure determination of Cu(BpT)Br***

X-ray crystallographic data of Cu(BpT)Br compound was collected on a Bruker SMART Apex II CCD diffractometer using graphite-monochromated Mo−Kαradiation(*λ* = 0.71073 Å). The structure of Cu(II) compound was solved by direct methods and refined against *F*^2^ by full-matrix least-squares methods using the SHELXTL version 5.1 (Sheldrick, 1997). All of the non-hydrogen atoms were refined anisotropically. All H atoms were placed in geometrically ideal positions and constrained to ride on their parent atoms. The crystallographic data for Cu(BpT)Br compound is summarized in Table S2. Selected bond lengths and angles are given in Table S3.

***1.2 Cytotoxicity assay (MTT)***

The MCF-7/ADRcells, MCF-7 cellsand WI-38cells were used to evaluate theanticancer activity of HSA-NAMI-A-Cu(BpT)Br-DOX complex. The free metallodrugs (NAMI-A and Cu(BpT)Br) and DOX, and the mixture of NAMI-A/Cu(BpT)Br/DOX (molar ratio 1:1:1) were used as control.One hundred microliters of cell suspensions at a density of 5 × 10^4^ cells/mL was seeded in triplicate in 96-well plates and incubated for 24 h at 37 °C in 5% CO_2_. Then the medium was replaced with the respective medium with 10% FBS containing different formulations at various concentrations and incubated at 37 °C under conditions of 5% CO_2_ for 48 h. The absorbance was read by enzyme labeling instrument with 570/630 nm double wavelength measurement. The cytotoxicity was evaluated based on the percentage of cell survival compared with the negative control. All of the tests were repeated in at least three independent experiments.

***1.3 Western blot analysis***

MCF-7/ADR cells were seeded into 3.5 cm dishes for 24 h, and then exposed to the HSA-NAMI-A-Cu(BpT)Br-DOX at indicated concentrations for 24 h. Cells were harvested and washed with ice-cold PBS three times, then they were lysed in radioimmunoprecipitation assay (RIPA) buffer. The protein concentration of the supernatant was determined by BCA (bicinchoninic acid) assay. Equal amounts of cellular total proteins were separated on SDS−polyacrylamide gel electrophoresis (10%) and then transferred onto poly(vinylidenedifluoride) membranes (Millipore, MA, USA) and blocked with 5% nonfat milk in TBST buffer (20 mMTris, pH 8.0, 150 mMNaCl, and 0.05% Tween 20) for 1 h. Then, the membranes were incubated with the primary antibodies overnight at 4 °C. After a subsequent washing step, the membrane is incubated with the appropriate secondary antibodies conjugated with horseradish peroxidase for 1 h at room temperature and washed for three times with TBST. The immunoreactivity was detected using Amersham ECL Plus (Amersham) western blotting detection reagents.

**2 Structure of Cu Compound**

The structure of Cu(BpT)Br crystallizes in the monoclinic system with space group*C*2/*c*. As shown in Figure S3, the Cu(II) center exists in a slightly distorted square planer geometry with thiosemicarbazone BpT^−^ acting as a tridentate ligand. Thebromine ion, *trans* to the carboxamido nitrogen (N3), completes coordinationin the square plane. Here, the Cu−N(pyridine) distance [Cu1−N4 = 2.005Å] is relatively longer than the Cu−N(amido) distance [Cu1−N3 = 1.989Å], indicating the different strength of thebond formed by each of the coordinated nitrogen atoms. In addition, the C–S bonds (C1−S1) of 1.734 Åin the compound was intermediate between the formal single and double bonds, indicating that the thiocarbonyl moiety in the Cu(II)compound adopted a tautomeric form and acted as a mono-negative (mercapto group) ligand, which support thiolate formation in the ligands on complexation.

In the solid state,the monomeric units of Cu(BpT)Br were arranged in a dimer fashion such that the coordinated Br of one monomeric unit stacks on thenext one to axially interact with the neighboring copper(II)center. The dimers were further linked into a one-dimensional (1D) polymeric chain by the N−H···N hydrogen bondsinvolving a nitrogen atom (N1) from the BpT ligand and a N2^i^ from the neighboring BpT ligand (N1···N2^i^ = 3.029 Å and the N1−H1B···N2^i^ angle is 179.1º, symmetry code: (i) −*x*, 1 −*y*, 2−*z*, Figure S4).

**References**

Sheldrick GM. (1997). *SHELXTLV5.1, Software, Reference, Manual*., BrukerAXS, Inc, Madison, W.I. U.S.A.

**Table S1** Data collection statistics and crystallographic analysis of HSA-NAMI-A-Cu(BpT)Br complex

| Data collection  Space group  Cell parameters, *a, b, c* (Å)  Cell parameters,  Resolution range (Å)  Data redundancy  Completeness (%) ^a^  *I*/σ  *R*_merge_ (%) ^b^  Model refinement  *R*_model_ (%) ^c^  *R*_free_ (%) ^d^  r.m.s. deviation from ideal bond lengths  r.m.s. deviation from ideal angles (°) | *P*1  96.02, 95.16, 38.76  75.22, 89.79, 78.74  50−2.6  4.3  96% (98.8%)  14.2 (4.4)  6.6% (24.1%)  18.53%  24.55%  0.009 Å  1.170 |
| --- | --- |

^a^ Values for the outermost resolution shell are given in parentheses.

^b^ R_merge_=100×Σ_h_Σ_j_| I_hj_-I_h_|/Σ_h_Σ_j_ I_hj_ where I_h_ is the weighted mean intensity of the symmetry-related refractions I_hj_.

^c^ R_model_=100×Σ_hkl_|F_obs_ -F_calc_|/Σ_hkl_F_obs_ where F_obs_ and F_calc_ are the observed and calculated structure factors, respectively.

^d^ R_free_ is the R_model_ calculated using a randomly selected 5% sample of reflection data omitted from the refinement.

**Table S2** Crystal data for Cu(BpT)Br compound.

| Complex | Cu(BpT)Br |
| --- | --- |
| Empirical formula | C_13_H_11_BrCuN_4_S |
| Molecular weight | 398.77 |
| Crystal system | monoclinic |
| Space group | *C*2*/c* |
| *a* (Å) | 11.0879(16) |
| *b* (Å) | 22.369(3) |
| *c* (Å) | 12.1279(18) |
| *α* (^o^) | 90.00 |
| *β* (^o^) | 105.075(2) |
| *γ* (^o^) | 90.00 |
| *T* (K) | 296.15 |
| *V* (Å^3^) | 2904.6(7) |
| *Z* | 8 |
| *ρ*_calc._ (g·cm^-3^) | 1.824 |
| *F*(000) | 1576 |
| *µ*(Mo-K_α_) (mm^-1^) | 4.392 |
| Data/restraint/parameters | 2974/0/186 |
| Goodness-of-fit on *F*^2^ | 1.000 |
| Final *R*_1_, *wR*_2_ [*I* > 2*σ*(*I*)] | 0.0258, 0.0638 |

**Table S3** Selected bond lengths [Å] and angles [º] in [Cu(BpT)Br]compound.

| Cu1−Br1 | 2.3925(5) | S1−Cu1−Br1 | 95.83(2) |
| --- | --- | --- | --- |
| Cu1−S1 | 2.2251(7) | S1−Cu1−N3 | 84.33(5) |
| Cu1−N3 | 1.9889(17) | N4−Cu1−N3 | 80.47(7) |
| Cu1−N4 | 2.005(2) | N4−Cu1−Br1 | 99.36(5) |
| S1−C1 | 1.734(2) | N4−Cu1−S1 | 164.22(6) |
| Br1−Cu1−N3 | 179.72(6) |  |  |


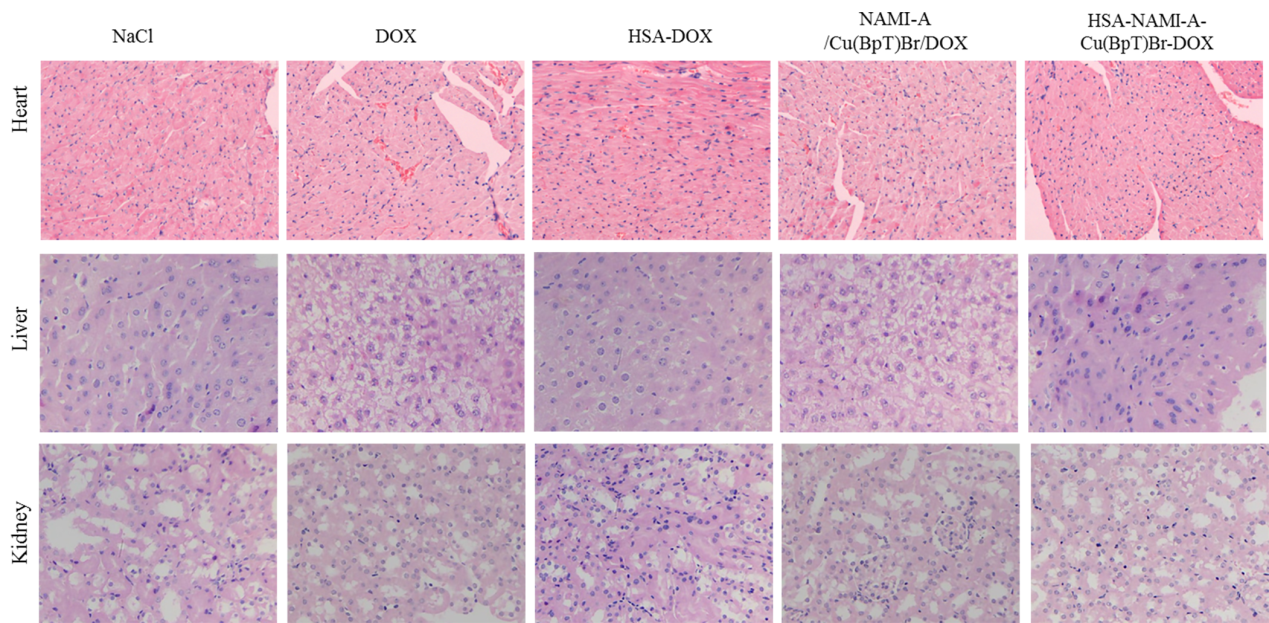


**Figure S1** H&E staining analysis of organs sections treated with various treatments (magnification ×400).


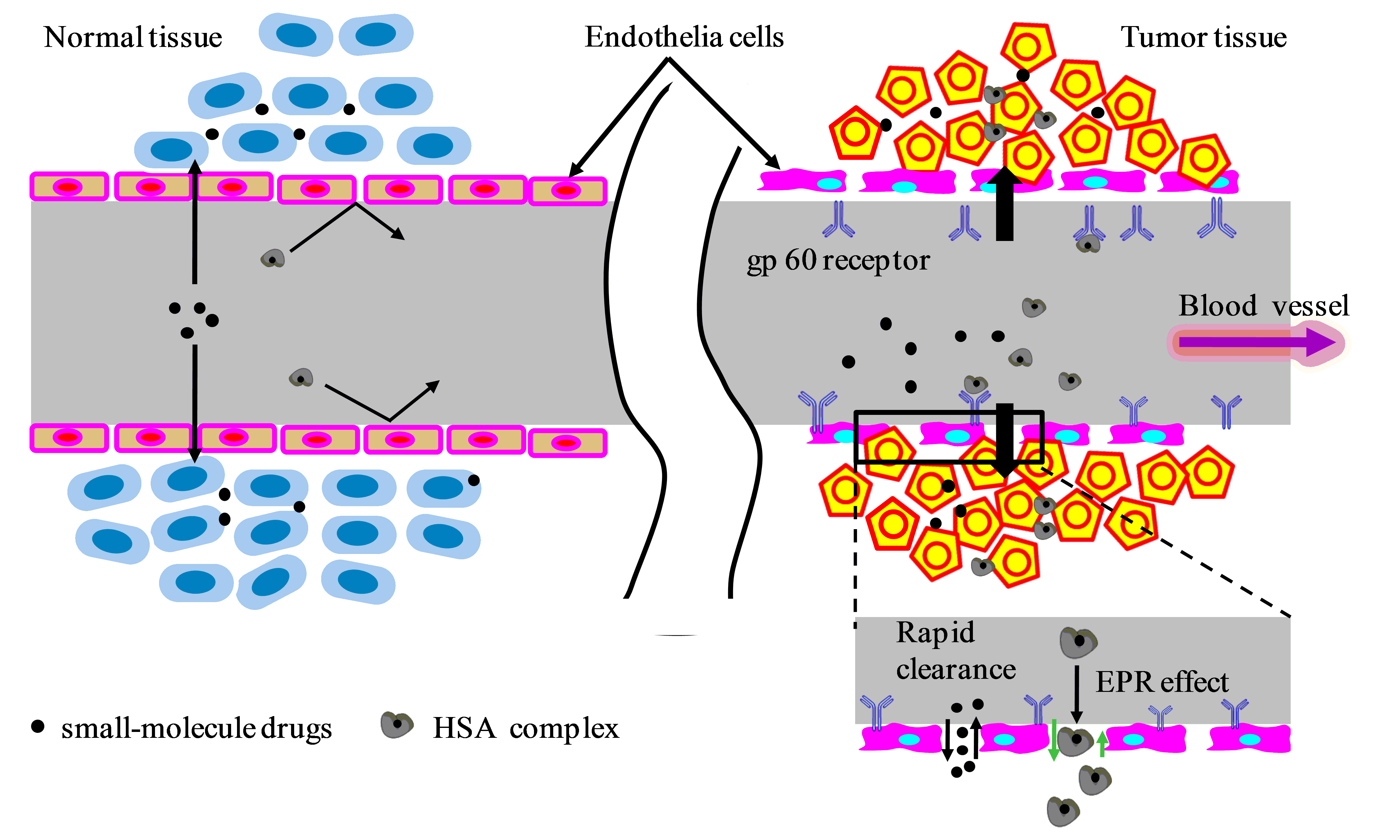


**Figure S2** Transport of HSA complex and small-molecule drugs through normal (left) and tumor (right) tissues. The enhanced permeability and retention (EPR) effect is a unique feature of tumors, allowing HSA complex to accumulate more in tumor tissues than in normal tissues.


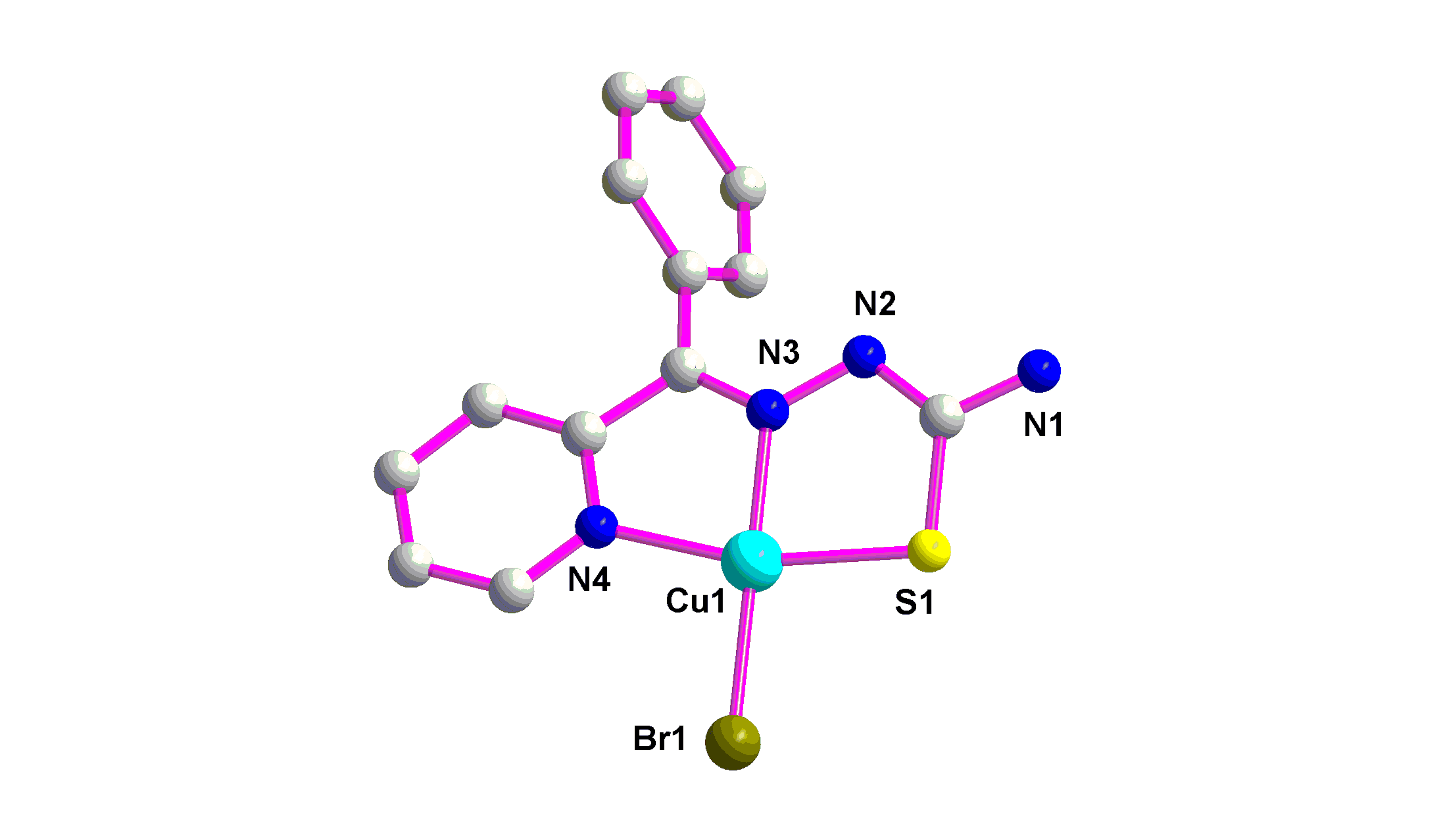


**Figure S3** The local coordination environment of[Cu(BpT)Br] compound.


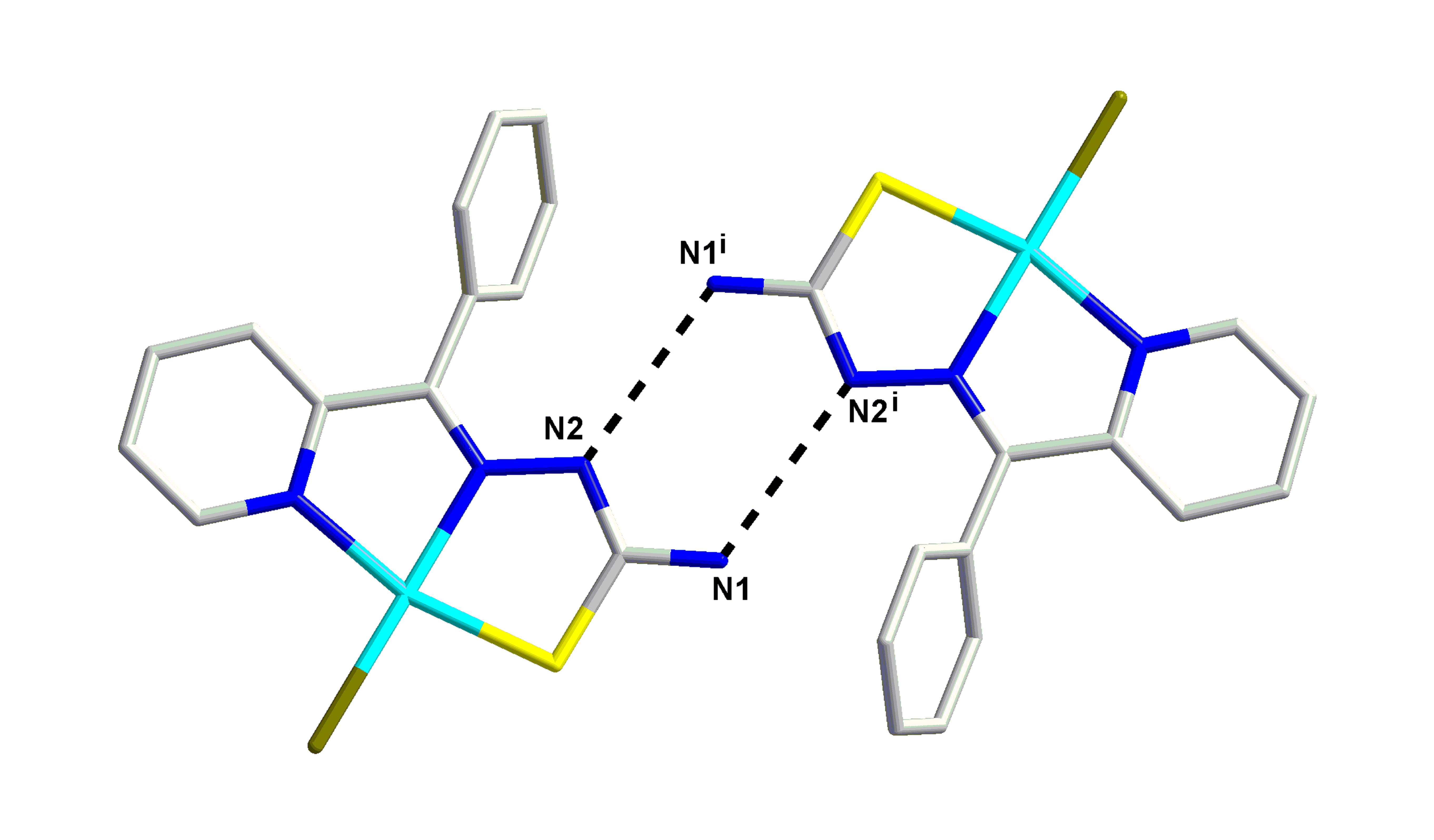


**Figure S4** A dimer fashion formed by the N−H···N interacting in Cu(II) compound (Symmetry code: (i) – *x*, 1 – *y*, 2 – *z*).
